# Supplementary material for: Investigation of the effects of probiotic, Bacillus subtilis on stress reactions in laying hens using infrared thermography
Source: PLoS One. 2020 Jun 11;15(6):e0234117. doi: 10.1371/journal.pone.0234117 (PMC7292008; doi:10.1371/journal.pone.0234117)
Supplement: Attachment — Submitted filename: Response to Reviewers.docx [file pone.0234117.s001.docx]

**Responses to reviewers' comments, manuscript Ref: PONE-D-20-10311, entitled “Investigation of the effects of probiotic, Bacillus subtilis on stress reactions in laying hens using infrared thermography”.**

**Comments to the Author**

1. **Comment:** Please ensure that your manuscript meets PLOS ONE's style requirements, including those for file naming. The PLOS ONE style templates can be found at:

https://journals.plos.org/plosone/s/file?id=wjVg/PLOSOne_formatting_sample_main_body.pdf and

https://journals.plos.org/plosone/s/file?id=ba62/PLOSOne_formatting_sample_title_authors_affiliations.pdf

**Response**: The article style according to journal requirements has been checked and corrected.

2. **Comment**: Thank you for stating the following financial disclosure:

"No"

At this time, please address the following queries:

a. Please clarify the sources of funding (financial or material support) for your study. List the grants or organizations that supported your study, including funding received from your institution.

**Response:** The authors received no specific funding for this work. Maria Soroko was supported by Fulbright Scholarship (Fulbright Senior Award) to conduct thermographic examination on birds at Purdue University. Rest of the research on poultry was supported by the USDA National Institute of Food and Agriculture, Foundation program of the Agriculture and Food Research Initiative Competitive Grants Program under the Award No: 2017-67015-26567. Mention of trade names or commercial products in this article is solely for the purpose of providing specific information and does not imply recommendation or endorsement of the USDA. The USDA is an equal opportunity provider and employer.

b. State what role the funders took in the study. If the funders had no role in your study, please state: “The funders had no role in study design, data collection and analysis, decision to publish, or preparation of the manuscript.”

**Response:** The funders had no role in study design, data collection and analysis, decision to publish, or preparation of the manuscript.

c. If any authors received a salary from any of your funders, please state which authors and which funders.

**Response:** None of the authors received any financial support.

d. If you did not receive any funding for this study, please state: “The authors received no specific funding for this work.”

**Response:** The authors received no specific funding for this work.

e. Please include your amended statements within your cover letter; we will change the online submission form on your behalf.

**Response:** Amended statements have been added in the cover letter.

3. **Comment:** Thank you for stating the following in your Competing Interests section:

"No"

a) Please complete your Competing Interests statement to state any Competing Interests. If you have no competing interests, please state "The authors have declared that no competing interests exist.", as detailed online in our guide for authors at <http://journals.plos.org/plosone/s/submit-now>
b) This information should be included in your cover letter; we will change the online submission form on your behalf.

**Response:** The authors have declared that no competing interests exist. The information has been included in the cover letter.

Please know it is PLOS ONE policy for corresponding authors to declare, on behalf of all authors, all potential competing interests for the purposes of transparency. PLOS defines a competing interest as anything that interferes with, or could reasonably be perceived as interfering with, the full and objective presentation, peer review, editorial decision-making, or publication of research or non-research articles submitted to one of the journals. Competing interests can be financial or non-financial, professional, or personal. Competing interests can arise in relationship to an organization or another person. Please follow this link to our website for more details on competing interests: http://journals.plos.org/plosone/s/competing-interests

**Reviewers' comments:**

Is the manuscript technically sound, and do the data support the conclusions?

The manuscript must describe a technically sound piece of scientific research with data that supports the conclusions. Experiments must have been conducted rigorously, with appropriate controls, replication, and sample sizes. The conclusions must be drawn appropriately based on the data presented.

Reviewer #1: Yes

Reviewer #2: Yes

Has the statistical analysis been performed appropriately and rigorously?

Reviewer #1: Yes

Reviewer #2: Yes

Have the authors made all data underlying the findings in their manuscript fully available?

The PLOS Data policy requires authors to make all data underlying the findings described in their manuscript fully available without restriction, with rare exception (please refer to the Data Availability Statement in the manuscript PDF file). The data should be provided as part of the manuscript or its supporting information, or deposited to a public repository. For example, in addition to summary statistics, the data points behind means, medians and variance measures should be available. If there are restrictions on publicly sharing data—e.g. participant privacy or use of data from a third party—those must be specified.

Reviewer #1: Yes

Reviewer #2: Yes

Is the manuscript presented in an intelligible fashion and written in standard English?

PLOS ONE does not copyedit accepted manuscripts, so the language in submitted articles must be clear, correct, and unambiguous. Any typographical or grammatical errors should be corrected at revision, so please note any specific errors here.

Reviewer #1: Yes

Reviewer #2: Yes

**Review Comments to the Author**

**Reviewer #1**: I have not found significant limitations; conversely, I think that this manuscript has many strengths, such as originality and methodological accuracy. I think that this paper I reviewed reports novel findings useful for poultry health and wellbeing. The methods, results and data interpretation meet the journal's standard; no errors have been detected and the conclusions have been properly supported. The trials were well described and the findings have been well discussed. Moreover, the language is also fine.

**Reviewer #2:** The manuscript investigates the use of a probiotic in laying hens as a way to reduce stress. To evaluate this stress, infrared thermography was used. The manuscript uses good approaches and is acceptable with regard to content. The manuscript concludes that probiotics did not reduce stress under the conditions evaluated. However, infrared thermography could be used to evaluate stress in laying hens.

1. **Comment:** Page 2, line 24-27: Why was a beak trimmed group included in the study? Should there have been a beak trimmed with probiotics to effectively compare?

**Response:** Thank you for your comment. We included beak trimmed group fed regular diet because according to previous studies beak trimming reduces stress response in birds. Therefore we wanted to indicate if there are any temperature changes between beak trimmed – fed regular diet, non-beak trimmed fed a regular diet; and non-beak trimmed fed a diet supplemented with probiotics. Future research could include temperature differences between: beak trimmed fed a diet supplemented with probiotics and beak trimmed fed a regular diet (as a control group).

2. **Comment:** The abstract reads that ”Seventy-one white and 70 brown hens were used.” But page 6, line 109 reads, “396 female chicks of each line” were housed. I assume this is the starting number of conditioned/pullets that will be selected from for the study.

**Response:** Thank you for your comment. I confirm 396 female chicks of each line that was a starting number and in the research we included a part od those birds n = 141 of both lines.

3. **Comment:** To clarify the materials and methods, 141 birds were broken into 3 groups which will break the groups into 47 birds each. This would be further broken down into 23-24 Hyline W-36 hens and 23-24 Brown hens per group.
   **Response**: Thank you for that comment: 141 bird included 2 lines: 71 Hy-Line W-36 hens and 70 Brown hens. Each line was broken into 3 groups: including 71 Hy-Line W-36 hens (group G - 24 birds, B - 24 birds, and Y- 23 birds) and 70 Brown hens (group G’ - 23 birds, B’- 24 birds and Y’- 23 birds). More information about groups has been added at lines: 132-133 page 7 and 8.
4. **Comment:** Page 10, line 171-173: Results. The beak trimmed birds and non-beaked trimmed were pooled for further analysis. How were these pooled with the other groups? Were they included in the non-probiotic groups or added to both of the other groups? Please clarify.
   **Response**: Thank you for very much your comment. This sentence is superfluous. It has been delete from the manuscript.
5. **Comment**: Is there a significance for the references that were in red or were these corrections?

**Response**: Thank you for pointing this. There was no reason for highlighting references, it was done by mistake.
